# A sticky situation: extracellular DNA shapes *Aspergillus fumigatus* biofilms

**DOI:** 10.3389/fmicb.2013.00159

**Published:** 2013-06-28

**Authors:** Sven Krappmann, Gordon Ramage

**Affiliations:** ^1^Microbiology Institute - Clinical Microbiology, Immunology and Hygiene, University Hospital of Erlangen, Friedrich-Alexander-University Erlangen-NürnbergErlangen, Germany; ^2^School of Medicine, College of Medical, Veterinary and Life Sciences, University of GlasgowGlasgow, UK

Multicellular stages play an important role for microbial communities as well as during infections. For one of the major fungal pathogens, the environmental mold *Aspergillus fumigatus*, an interesting and relevant morphotype beyond the conidial and hyphal stages has gained increasing attention: the multicellular biofilm. Such hyphal communities were first described for *A. fumigatus* in 2007 (Mowat et al., 2007) and are nowadays recognized to be relevant in the clinic (Müller et al., 2011).

*A. fumigatus* is environmentally omnipresent and its airborne conidia are constantly inhaled to become eliminated by the functional host immune system. In case of immune response failure, these infectious propagules germinate and invade the infected tissue to cause forms of aspergillosis (Dagenais and Keller, 2009). Treatment options for this disease are still inadequate, based on the limited number of antifungal drugs and their side effects. Resistance of *A. fumigatus* to antifungal treatment is influenced by several factors, among them biofilm formation (Seidler et al., 2008). In these structures, a dense network of hyphae becomes embedded in an extracellular matrix (ECM) that is mainly composed of polysaccharides. The ECM of such biofilms grown *in vitro* further contains melanin, proteins like hydrophobins or antigens, polyols, and monosaccharides (Beauvais et al., 2007). *A. fumigatus* biofilms are also formed *in vivo* during invasive aspergillosis or aspergilloma and have been characterized to slightly differ from their *in vitro* counterparts with respect to ECM composition (Loussert et al., 2010).

Recent evidence has identified extracellular DNA (eDNA) as important component of *A. fumigatus* biofilms, either derived from fungal autolysis (Rajendran et al., 2013) or, as elegantly demonstrated in a recent publication by Brakhage, Hillmann, and colleagues, when externally supplied (Shopova et al., 2013). Probably supported by a high affinity of nucleic acids to the cell wall of *A. fumigatus* (Jöchl et al., 2009) that is based on electrostatic interactions, extrinsic eDNA promotes adhesion of the conidia in the initial phase of biofilm formation, triggers polysaccharide formation and becomes incorporated in the biofilm, thereby shaping its overall structure. Accordingly, DNA contributes to the structural integrity of mature *A. fumigatus* biofilms, a finding that has been described for several bacterial biofilm formers before (Flemming and Wingender, 2010) and that highlights the structural conservation of microbial biofilms. Considering that *A. fumigatus* commonly colonizes patients suffering from cystic fibrosis and that the mucus of such patients contains extrinsic DNA at high concentrations that may stem from of neutrophil extracellular traps (NETs) (Brinkmann et al., 2004; Bruns et al., 2010), which are released by a special form of cell death termed NETosis (Remijsen et al., 2011; Almyroudis et al., 2013), from necrotic tissue, or from competing microorganisms, the formation of biofilms is of special significance in this particular host group.

In the light of the clinical as well as industrial relevance of *Aspergillus* biofilms (Ramage et al., 2011), the newly presented data and insights not only sharpen our image of this multicellular stage within the *Aspergillus* lifestyle but also provide perspective for improvement and new avenues in antifungal therapy or biotechnological applications.

## References

[B1] Almyroudis N. G. Grimm M. J. Davidson B. A. Röhm M. Urban C. F. Segal B. H. (2013). NETosis and NADPH oxidase: at the intersection of host defense, inflammation, and injury. Front. Immunol. 4:45 10.3389/fimmu.2013.00045 23459634 PMC3585429

[B2] Beauvais A. Schmidt C. Guadagnini S. Roux P. Perret E. Henry C. (2007). An extracellular matrix glues together the aerial-grown hyphae of *Aspergillus fumigatus*. Cell. Microbiol. 9, 1588–1600 10.1111/j.1462-5822.2007.00895.x 17371405

[B3] Brinkmann V. Reichard U. Goosmann C. Fauler B. Uhlemann Y. Weiss D. S. (2004). Neutrophil extracellular traps kill bacteria. Science 303, 1532–1535 10.1126/science.1092385 15001782

[B4] Bruns S. Kniemeyer O. Hasenberg M. Aimanianda V. Nietzsche S. Thywissen A. (2010). Production of extracellular traps against *Aspergillus fumigatus in vitro* and in infected lung tissue is dependent on invading neutrophils and influenced by hydrophobin RodA. PLoS Pathog. 6:e1000873 10.1371/journal.ppat.1000873 20442864 PMC2861696

[B5] Dagenais T. R. Keller N. P. (2009). Pathogenesis of *Aspergillus fumigatus* in invasive Aspergillosis. Clin. Microbiol. Rev. 22, 447–465 10.1128/CMR.00055-08 19597008 PMC2708386

[B6] Flemming H. C. Wingender J. (2010). The biofilm matrix. Nat. Rev. Microbiol. 8, 623–633 10.1038/nrmicro2415 20676145

[B7] Jöchl C. Loh E. Ploner A. Haas H. Hüttenhofer A. (2009). Development-dependent scavenging of nucleic acids in the filamentous fungus *Aspergillus fumigatus*. RNA Biol. 6, 179–186 10.4161/rna.6.2.7717 19246986

[B8] Loussert C. Schmitt C. Prevost M. C. Balloy V. Fadel E. Philippe B. (2010). *In vivo* biofilm composition of *Aspergillus fumigatus*. Cell. Microbiol. 12, 405–410 10.1111/j.1462-5822.2009.01409.x 19889082

[B9] Mowat E. Butcher J. Lang S. Williams C. Ramage G. (2007). Development of a simple model for studying the effects of antifungal agents on multicellular communities of *Aspergillus fumigatus*. J. Med. Microbiol. 56, 1205–1212 10.1099/jmm.0.47247-0 17761484

[B10] Müller F. M. Seidler M. Beauvais A. (2011). *Aspergillus fumigatus* biofilms in the clinical setting. Med. Mycol. 49 Suppl. 1, S96–S100 10.3109/13693786.2010.502190 21254964

[B11] Rajendran R. Williams C. Lappin D. F. Millington O. Martins M. Ramage G. (2013). Extracellular DNA release acts as an antifungal resistance mechanism in mature *Aspergillus fumigatus* biofilms. Eukaryot. Cell 12, 420–429 10.1128/EC.00287-12 23314962 PMC3629765

[B12] Ramage G. Rajendran R. Gutierrez-Correa M. Jones B. Williams C. (2011). *Aspergillus* biofilms: clinical and industrial significance. FEMS Microbiol. Lett. 324, 89–97 10.1111/j.1574-6968.2011.02381.x 22092808

[B13] Remijsen Q. Kuijpers T. W. Wirawan E. Lippens S. Vandenabeele P. Vanden Berghe T. (2011). Dying for a cause: NETosis, mechanisms behind an antimicrobial cell death modality. Cell Death Differ. 18, 581–588 10.1038/cdd.2011.1 21293492 PMC3131909

[B14] Seidler M. J. Salvenmoser S. Müller F. M. (2008). *Aspergillus fumigatus* forms biofilms with reduced antifungal drug susceptibility on bronchial epithelial cells. Antimicrob. Agents Chemother. 52, 4130–4136 10.1128/AAC.00234-08 18710910 PMC2573142

[B15] Shopova I. Bruns S. Thywissen A. Kniemeyer O. Brakhage A. A. Hillmann F. (2013). Extrinsic extracellular DNA leads to biofilm formation and colocalizes with matrix polysaccharides in the human pathogenic fungus *Aspergillus fumigatus*. Front. Microbiol. 4:141 10.3389/fmicb.2013.00141 PMC3674311 23760756

